# Speech-in-noise perception ability can be related to auditory efferent pathway function: a comparative study in reading impaired and normal reading children

**DOI:** 10.1016/j.bjorl.2018.11.010

**Published:** 2019-01-28

**Authors:** Mehdi Akbari, Rasool Panahi, Ayub Valadbeigi, Morteza Hamadi Nahrani

**Affiliations:** Iran University of Medical Sciences, School of Rehabilitation Sciences, Department of Audiology, Tehran, Iran

**Keywords:** Medial olivocochlear bundle, Otoacoustic emission, Speech-in-noise perception, Reading impairment, Feixe olivococlear medial, Emissão otoacústica, Percepção de fala no ruído, Dificuldade de leitura

## Abstract

**Introduction:**

Deficient auditory processing can cause problems with speech perception and affect the development and evolution of reading skills. The efferent auditory pathway has an important role in normal auditory system functions like speech-in-noise perception, but there is still no general agreement on this.

**Objective:**

To study the performance of the efferent auditory system in a group of children with reading impairment in comparison with normal reading and evaluation of its relationship with speech-in-noise perception.

**Methods:**

A total of 53 children between the ages of 8–12 years were selected for the study of which 27 were with reading impairment and 26 were normal reading children. Transient evoked otoacoustic emissions suppression and auditory recognition of words-in-noise test were performed for all the children.

**Results:**

The average amplitude of transient evoked otoacoustic emissions suppression showed a significant difference between the two groups in the right (*p* = 0.004) and in the left ear (*p* = 0.028). Assessment of the relationship between transient evoked otoacoustic emissions suppression and monaural auditory recognition of words-in-noise scores showed a significant moderate negative relationship only in the right ear (*p* = 0.034, *r* = −0.41) of the normal reading children. Binaural auditory recognition of words-in-noise scores were significantly correlated with the amplitude of transient evoked otoacoustic emissions suppression in the right ear (*p* < 0.001, *r* = −0.75) and in the left ear (*p* < 0.001, *r* = −0.64) of normal reading children. In the reading impaired group, ?a weaker correlation was observed between binaural auditory recognition of words-in-noise scores and transient evoked otoacoustic emissions suppression in the right (*p* = 0.003, *r* = −0.55) and in the left ear (*p* = 0.012, *r* = −0.47).

**Conclusions:**

Transient evoked otoacoustic emissions suppression pattern in the reading impaired group was different compared with normal reading children, and this difference could be related to efferent system performance. Words-in-noise scores in children with impaired reading were lower than in normal reading children. In addition, a relationship was found between transient evoked otoacoustic emissions suppression and words-in-noise scores in both normal and impaired reading children.

## Resumo

**Introdução:**

O processamento auditivo deficiente pode causar problemas na percepção da fala e afetar o desenvolvimento e a evolução das habilidades de leitura. A via auditiva eferente tem um papel importante nas funções do sistema auditivo normal, como a percepção da fala no ruído, mas ainda não há um consenso sobre isso.

**Objetivo:**

Estudar o desempenho do sistema auditivo eferente em um grupo de crianças com dificuldade de leitura em comparação às com leitura normal e avaliação de sua relação com a percepção da fala no ruído.

**Método:**

Foram selecionadas para o estudo 53 crianças entre oito e 12 anos, das quais 27 tinham dificuldade de leitura e 26 apresentavam leitura normal. A avaliação por emissões otoacústicas evocadas transientes e o teste *auditory recognition of words-in-noise* foram feitos em todas as crianças.

**Resultados:**

A amplitude média da supressão das emissões otoacústicas evocadas transientes mostrou diferença significante entre os dois grupos na orelha direita (*p* = 0,004) e esquerda (*p* = 0,028). A avaliação da relação entre a supressão das emissões otoacústicas evocadas transientes e os escores monoaurais do teste *auditory recognition of words-in-noise* mostrou uma relação negativa moderadamente significante apenas na orelha direita (*p* = 0,034, r = -0,41) das crianças com leitura normal. Os escores binaurais do *auditory recognition of words-in-noise* foram significantemente correlacionados com a amplitude de supressão das emissões otoacústicas evocadas transientes na orelha direita (*p* < 0,001, r = -0,75) e na orelha esquerda (*p* < 0,001, r = -0,64) das crianças com leitura normal. No grupo com dificuldade de leitura, uma correlação mais fraca foi observada entre os escores binaurais do *auditory recognition of words-in-noise* e supressão das emissões otoacústicas evocadas transientes, na orelha direita (*p* = 0,003, r = -0,55) e na esquerda (*p* = 0,012, r = -0,47).

**Conclusões:**

O padrão de supressão das emissões otoacústicas evocadas transientes no grupo com dificuldade de leitura foi diferente em comparação com as crianças com leitura normal e essa diferença pode estar relacionada ao desempenho do sistema eferente. Os escores de palavras no ruído em crianças com dificuldade de leitura foram menores do que nas crianças com leitura normal. Além disso, foi encontrada uma relação entre a supressão das emissões otoacústicas evocadas transientes e os escores de palavras no ruído tanto em crianças com leitura normal quanto nas com dificuldade de leitura.

## Introduction

Many children develop fluent reading ability during primary school, while about 5–12% of them have difficulty learning to read in the absence of sensory or cognitive problems with regards to normal intelligence and adequate education.^1^ Problems with learning to read are termed “specific reading disability” or “dyslexia”.^2^ Fluent reading requires adequate language perception and fluent word recognition.^2^ Many studies have identified deficient speech perception in individuals with learning disorders and reading impairment.^3^, ^4^, ^5^, ^6^ It appears that speech perception problems in these groups are related to phonological processing.^5^ A normal auditory system is one of the requirements for the acquisition of normal language and phonological abilities.^7^ It has been suggested that deficient auditory processing can cause problems with speech perception, and as such, affect the development and evolution of reading skills.^8^ Investigations of children with Learning Disability (LD) have indicated that auditory processing of speech can be abnormal at the brainstem level^9^ and it is as though this problem increases when background noise is added. From previous studies, it can be inferred that generally individuals with learning disorders such as dyslexia have problems with speech in noise perception. However, there is no consensus on this issue as there are studies that did not report such findings.^10^

The central auditory system has a top-down effect on the lower levels through the efferent auditory pathway which terminates at the cochlea. It has been suggested that the efferent auditory pathway contributes to several aspects such as cochlea protection, vowel discrimination, and processing of complex auditory signals.^11^, ^12^ The Superior Olivocochlear Complex (SOC) connects with the ipsilateral and contralateral cochlea through olivocochlear bundle fibers. The contralateral fibers of the efferent system are known as the Medial Olivocochlear Bundle (MOCB) and are mostly connected to outer hair cells.^11^ With the introduction of Otoacoustic Emissions (OAEs), the evaluation of efferent auditory system function has become possible through simultaneous recording of OAEs and contralateral wide-band noise presentation. Therefore, it is possible to use a non-invasive and fast method using measurements of OAEs suppression to assess efferent auditory system function, especially MOCB.^13^, ^14^, ^15^

Many researchers have suggested that efferent auditory system activity can improve speech perception performance in the presence of background noise, and this improved performance could be observed even in situations where there is no background noise.^16^, ^17^, ^18^, ^19^ It is assumed that the auditory efferent system improves the signal to noise ratio through an “anti-masking” effect. This means that cochlear amplifier gain will be reduced as a result of MOCB activation. Therefore, noise-induced neural adaptation will be decreased and thus improves our ability to detect signal and overcome competitive noise.^20^, ^21^ However, some investigations have reported no relationship between efferent auditory system function and speech perception in noise.^22^, ^23^ Some types of OAEs that are sensitive to cochlear function are Transient Evoked Otoacoustic Emissions (TEOAEs).^24^ If the peripheral auditory system is normal, abnormal changes in the TEOAE amplitude in response to contralateral noise can be considered as an abnormality of MOCB function. Here, with regard to studies that address the abnormal function of the efferent auditory system in auditory processing disorders and learning disabilities,^9^, ^13^, ^25^ it is thus hypothesized that efferent auditory system function varies in children with RI compared to NR children and that there is a relationship between speech-in-noise perception ability and auditory efferent pathway function. Most of the previous research has investigated the relationship between MOCB activity and speech-in-noise performance at particular Signal-to-Noise Ratios (SNRs). However, since the effects of MOCB function are more pronounced in the more challenging conditions,^17^ in the present study, instead of using of a particular SNR, we tried to calculate the smallest SNRs required for discrimination of a particular number of words and to study its association with TOAEs suppression amplitude.

## Material and methods

### Subjects

Two groups of 8–12 years old participants, including reading impaired children and control group, were assessed. RI group consisted of 27 students (18 boys) with a mean age 9.8 ± 1.0 years. They were invited to participate in the study from primary schools for learning disabilities. In the NR group, we examined 26 NR students (17 boys) with a mean age 10.1 ± 1.1 years who were invited from ordinary primary schools. Inclusion criteria for both groups were: IQ level of 90 or higher in Wechsler Intelligence Scale for Children-Revised (3rd Edition) (WISCIII-R) test, Right-handed monolingual native Persian speakers, no sign of attention-deficit hyperactivity disorder or emotional problems, no previous records of repetitive ear infection or hearing loss, pure-tone thresholds equal or better than 20 dB HL at 250–8000 Hz octave frequencies, tympanogram Type A, acoustic reflex threshold equal or more than 70 dB HL in response to wideband noise stimulus (in order to avoid activation of the middle ear muscle reflex during recording of TEOAE responses). All of the children had normal TEOAE responses (response reproducibility of equal or more than 70% and SNR equal to or more than 6 dB). According to the school records for RI children, no one had other developmental disorders except reading impairment. The present study was registered under code number IR.IUMS.REC1395.9221303202 and approved by the ethics committee of Iran University of Medical Sciences.

### Evaluation tests

#### TEOAE suppression evaluation

Assessment of TEOAE was done using a calibrated Echoport ILO 292 OAE system product made by Otodynamics. We presented 280 linear click stimuli at 65 dB SPL intensity and averaged the responses. To perform the test, we used two separated OAE recordings in a passive listening condition: (1) Recording of TEOAEs when continues white noise being presented in the contralateral ear at 60 dB SPL; (2) Recording of the TEOAE responses without presentation of contralateral white noise. In each ear, the difference of the overall TEOAE response amplitude between two conditions was considered as suppression response.

#### Words-in-noise test

We used the Persian version of auditory recognition of words-in-noise test (ARWIN) which was originally developed by Wilson.^26^ The test has acceptable test–retest reliability for assessment of word recognition ability in noise.^27^, ^28^ This test includes three lists of 35 perceptually homogenized monosyllabic words which are recorded in the presence of multi-talker babble noise of six Persian speakers. We used List 1 for the right ear, List 2 for the left ear and List 3 for the binaural condition. As defined by Mahdavi et al.,^27^ the test was presented in an audiometric booth at 60 dB HL by connecting a compact disc player to an audiometer. Words were presented monaurally in descending order in different signal-to-noise ratios from +24 to 0 dB in 4 dB steps. Five monosyllable words were presented in each SNR level and the children were asked to repeat every word they hear. A short alerting statement “say” was placed before each item, to warn the test subject against oncoming test item. A total number of correctly recognized words at different SNR levels were calculated through ARWIN score sheets. The ARWIN determines the signal-to-noise ratio in dB in which 50% of words were recognized correctly. Before performing the tests, the method and the goals of the study were explained to the children and their parents and they asked to sign and complete the consent forms.

### Statistics

Test results were analyzed using the Statistical Package for Social Sciences (SPSS) 16.0. Descriptive statistics were presented as the mean and Standard Deviation (SD) values. We used the Kolmogorov–Smirnov test to determine if sample data have a normal distribution. Analytic statistics were done using independent samples *t*-test for inter-group comparisons, paired Samples *t*-test for intra-group comparisons and Spearman correlation for assessment of the relationship between the amplitude of TEOAE suppression and word recognition ability in noise. A *p*-value of <0.05 was determined to be statistically significant.

## Results

### TEOAE evaluation

Generally, in the NR group, the amplitude of TEOAE suppression was higher than children with RI in the right and left ears. In the NR group, mean TEOAE suppression amplitude in the right ear was higher than the left with a significant difference (*p* = 0.024). In children with RI, there was no significant difference between the average responses of the ears (*p* = 0.36). Comparison of the average amplitude of TEOAE suppression between the two groups showed a significant difference in the right ear (*p* = 0.004) and in the left ear (*p* = 0.028). The results of the amplitude of TEOAE suppression and words in noise perception ability are presented and compared in Table 1, Table 2.

**Table 1:** TEOAE suppression and words in noise test results in the right ear.

Group	Ear		Right		*p*-Value	*t*
	NR		RI			
	*N*	Mean ± SD	*N*	Mean ± SD		
OAEsup. (dB)	26	1.8 ± 0.7	27	1.2 ± 0.8	0.004	3.0
M.ARWIN dB (50%)	26	2.0 ± 1.0	27	4.5 ± 1.3	<0.001	−7.5
B.ARWIN dB (50%)	26	1.6 ± 0.9	27	3.8 ± 1.2	<0.001	−7.2

OAEsup, TEOAE suppression; M.ARWIN, monaural ARWIN; B.ARWIN, binaural ARWIN.

**Table 2:** TEOAE suppression and words in noise test results in the left ear.

Group	Ear		Left		*p*-Value	*t*
	NR		RI			
	*N*	Mean ± SD	*N*	Mean ± SD		
OAEsup. (dB)	26	1.7 ± 0.7	27	1.3 ± 0.6	0.028	2.2
M.ARWIN dB (50%)	26	2.1 ± 1.4	27	4.6 ± 1.4	<0.001	−6.2
B.ARWIN dB (50%)	26	1.6 ± 0.9	27	3.8 ± 1.2	<0.001	−7.2

OAEsup, TEOAE suppression; M.ARWIN, Monaural ARWIN; B.ARWIN, Binaural ARWIN.

### Words-in-noise test

As presented in Table 1, the ARWIN scores were significantly different between the two groups (*p* < 0.001) in both ears. Generally, NR children needed less SNR to understand 50% of words in noisy situations, and in the assessment of the association between TEOAE suppression and ARWIN scores, stronger relationships were observed in NR children.

**Figure 1:**
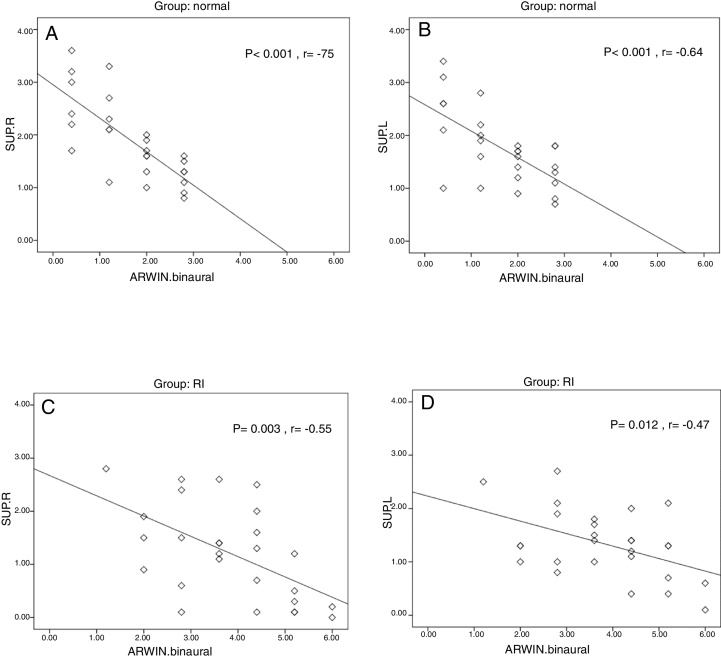
The relationship between the amplitude of TEOAE suppression and binaural ARWIN scores in the two groups.

In the NR group, assessment of the association between TEOAE suppression and ARWIN scores in the monaural ARWIN conditions showed a significant moderately negative relationship only in the right ear (*p* = 0.034, *r* = −0.41). In the binaural ARWIN condition, a significantly high negative correlation observed between ARWIN scores and the amplitude of TEOAE suppression in the right ear (*p* < 0.001, *r* = −0.75) and also a significantly moderate negative correlation in the left ear (*p* < 0.001, *r* = −0.64). In the RI group, no meaningful correlation was found between TEOAE suppression and the monaural ARWIN scores. In the binaural ARWIN condition, a significantly moderate negative correlation observed between ARWIN scores and the amplitude of TEOAE suppression in the right ear (*p* = 0.003, *r* = −0.55) and in the left ear (*p* = 0.012, *r* = −0.47). The correlation results for binaural ARWIN condition are presented in Fig. 1.

## Discussion

### TEOAE suppression amplitude difference and asymmetry

Due to lower levels of TEOAE suppression in children with auditory listening problems, it has been suggested that the efferent auditory system could play a role in learning in children with listening problems.^29^ Also, an abnormal efferent auditory pathway and decreased inhibition of OAEs have been reported in children with autism,^30^ Auditory Processing Difficulties (APD)^31^ and childhood selective mutism.^32^ In contrast, it has been reported that in learning disabled individuals, the presentation of noise in the contralateral ear may increase the amplitude of OAE response.^20^ In the case of reading-based learning disorders, it has been reported that contralateral suppression of DPOAEs in dyslexic children is insignificant in comparison with normal subjects.^33^ Significant differences in TEOAEs suppression in the right ear between two groups of normal children and children with poor school performance have been reported.^25^

Since language and reading skills formation in children depend on a healthy hearing system with functional integrity,^34^ it can be expected that children with fluent reading ability might have a better auditory function in comparison with the RI group. In the present study, the amplitude of TEOAE suppression in both ears in NR children was significantly larger than in children with RI. Decreased TEOAEs suppression amplitude in RI children represents abnormality in MOCB function. As previously mentioned, the efferent auditory pathway contributes to processing such as in vowel discrimination, and processing of complex auditory signals. If there is a deficit in auditory discrimination ability, one may not be able to relate what he/she hears (speech sounds) to what he/she sees (letters). This could result in the development of learning disorders.^35^

The present study investigated asymmetric suppression response between the two ears and revealed that there was a significant difference between them in the NR group, but no response asymmetry observed in the RI group. Researchers have shown that in various types of learning disorders, the asymmetry of hearing information processing at lower and higher levels can be different from that in normal people.^20^, ^36^, ^37^ As per asymmetry and MOCB activity between the two ears, this study agrees with previous researchers’ view of lateralization of the peripheral auditory system function and reduction of TEOAE amplitude in the right ear in response to contralateral noise.^38^, ^39^ TEOAEs asymmetry in normal individuals represents lateralization in the function of outer hair cells thus indicating that these cells can be more efficient or reactive in the right ear. The asymmetry of processing between the two ears also can provide useful binaural information to aid auditory input processing in the ear which has a higher ability for language processing.^38^ However, this finding is not consistent as indicated by different studies.^40^, ^41^ Based on the results of this and similar studies, one of the factors involved in irregular auditory processing asymmetry as observed in learning disorders can be attributed to efferent system dysfunction, including the auditory efferent pathway and MOCB.

### TEOAE suppression and speech-in-noise perception

Efferent auditory neurons play an important role in improving the detection of tones in the presence of background noise,^42^ increasing the ability to discriminate auditory stimulus intensity^43^ and speech perception in noise.^17^ However, there are some studies that did not present a clear relationship between the efferent auditory system and speech perception in noise.^22^, ^23^ The type of OAE response, the method used to determine OAE suppression value, the speech perception test material and the way in which the speech perception test is performed and rated might be the reasons for different results. In the present study, the relationship between TEOAE suppression and monaural ARWIN scores was shown to have a moderate negative correlation only in the right ear of the NR group. No considerable relationship was determined in the RI group. A negative correlation means that the higher the TEOAE suppression, the greater the ability to understand words in the presence of background noise. Therefore, normal-reading children were able to correctly identify 50% of the words in lower SNRs. The present study showed stronger speech perception in noise ability in the right ear of NR group, which could indicate a stronger relationship between the right ear and the left hemisphere and thus, a more prominent role of the right ear in the processing and understanding of speech in the presence of background noise. A finding was reported in which the sensory organs in the right ear were more sensitive to detect signal in the presence of noise because of the influence of efferent fibers from the left auditory cortex.^44^

In binaural ARWIN condition, higher correlation was observed between ARWIN scores and the amplitude of TEOAE suppression in both ears and both groups. Even in the RI group, a moderate negative correlation was observed. When interpreting these results, one must consider that in monaural ARWIN condition the target words and competing noise are presented simultaneously to the same ear. Ipsilateral presentation of noise would activate the ipsilateral MOCB reflex that is weaker than contralateral reflex.^45^ In the assessment of TEOAE suppression, we used contralateral noise that would activate both the ipsilateral and contralateral MOCB reflexes. Bilateral activation of MOCB reflex will result in stronger suppressive activity and stronger anti-masking effect than ipsilateral activation alone.^45^ Better speech-in-noise performance by presentation of contralateral stimulator has been reported previously.^17^, ^19^ Since the activation of both the ipsilateral and contralateral MOCB is much more likely to happen in binaural ARWIN condition, it is expected that the relationship between contralateral TEOAE suppression and ARWIN scores become more evident in binaural speech in noise test condition. In the present study in monaural ARWIN condition, a moderate relationship was observed only in the right ear of the NR group that could be related to its main connections with the left hemisphere. However, in binaural ARWIN condition higher correlation was observed between ARWIN scores and the amplitude of TEOAE suppression in both ears. In the RI group, the relationship was weaker than NR children. A dysfunction of the MOCB pathways and generally smaller amplitude of TEOAE suppression in the RI group were likely to prevent a stronger relationship being observed between words-in-noise recognition and amplitude of TEOAE suppression.

It is imperative to note that several neurobiological mechanisms in the bottom-up and top-down pathways are involved in speech perception in noise ability and thus cannot be limited to the function of MOCB and cochlea. However, it seems that the overall ability to understand speech in noise can be affected by the efferent auditory system function. Therefore, it can be concluded that the effects of the MOCB system are likely to assist in improving signal-to-noise ratio in NR individuals. The current finding is consistent with the hypothesis of the role of MOC efferent system in improving speech perception in noise. Similarly, to some extent, weaker speech perception in noise performance in the RI group can be attributed to a malfunction of the efferent auditory system. Such results can also provide more information on a pathophysiological background of reading impairment and provide support for the auditory processing deficit theory of dyslexia.

## Conclusions

The present study showed that the pattern of TEOAE suppression in the RI group was different from that of the NR group and thus this difference may be related to the function of the efferent system. Furthermore, this study showed that auditory recognition of words-in-noise in children with RI was weaker compared to NR children and that there was a reasonable correlation between TEOAE suppression and words-in-noise perception in NR children. However, in children with RI, the dysfunction of the MOCB pathways was likely to prevent a stronger relationship being observed between words-in-noise recognition and amplitude of TEOAE suppression.

## Conflicts of interest

The authors declare no conflicts of interest.
